# Changes in Defense of an Alien Plant *Ambrosia artemisiifolia* before and after the Invasion of a Native Specialist Enemy *Ophraella communa*

**DOI:** 10.1371/journal.pone.0049114

**Published:** 2012-11-07

**Authors:** Yuya Fukano, Tetsukazu Yahara

**Affiliations:** Department of Biology, Faculty of Science, Kyushu University, Fukuoka, Japan; Institute of Botany, Czech Academy of Sciences, Czech Republic

## Abstract

The evolution of increased competitive ability hypothesis (EICA) predicts that when alien plants are free from their natural enemies they evolve lower allocation to defense in order to achieve a higher growth rate. If this hypothesis is true, the converse implication would be that the defense against herbivory could be restored if a natural enemy also becomes present in the introduced range. We tested this scenario in the case of *Ambrosia artemisiifolia* (common ragweed) – a species that invaded Japan from North America. We collected seeds from five North American populations, three populations in enemy free areas of Japan and four populations in Japan where the specialist herbivore *Ophraella communa* naturalized recently. Using plants grown in a common garden in Japan, we compared performance of *O. communa* with a bioassay experiment. Consistent with the EICA hypothesis, invasive Japanese populations of *A. artemisiifolia* exhibited a weakened defense against the specialist herbivores and higher growth rate than native populations. Conversely, in locations where the herbivore *O. communa* appeared during the past decade, populations of *A. artemisiifolia* exhibited stronger defensive capabilities. These results strengthen the case for EICA and suggest that defense levels of alien populations can be recuperated rapidly after the native specialist becomes present in the introduced range. Our study implies that the plant defense is evolutionary labile depending on plant-herbivore interactions.

## Introduction

Human activities, such as migrations and global transportation, have spread many plant species beyond their native habitats. Some of these exotic plants have become invasive, seriously damaging native species and ecosystems [1], [2]. One of the explanations for the success of invasive plants is that the non-native environments may either be free of natural enemies or the presence of enemies may be significantly reduced. The result is enhanced fitness due to less damage from herbivory [3], [4]. This hypothesis is called the enemy release hypothesis (ERH) (Keane & Crawly 2002) and has been supported by comparing herbivory rates on invasive plants between their native and introduced ranges [5]–[7]. Yet, this hypothesis has not gone uncontested [8]–[11] and some studies have shown opposing evidence to suggest that invasive plants in the introduced range have suffered comparable or even higher herbivore pressure than in native relatives [8].

Blossey & Nötzold (1995) advanced this hypothesis by employing the optimal defense theory which assumes that plants in the conditions of limited resources will make a trade-off in resource allocation between growth, reproduction, storage, and defense [13]. They claimed that, in the absence of herbivores, natural selection favors more vigorous and competitive genotypes by reducing allocation to defense against native herbivores. This is the Evolution of Increased Competitive Ability (EICA) hypothesis and it predicts that (1) individuals with exotic genotypes show more vigor than individuals with native genotypes if compared under the same growing conditions, and (2) specialists from the home range will perform better on host plants with exotic genotypes. Recent studies supported the first prediction in some cases [14], [15], but did not support it in other cases [16], [17]. The second prediction also has yeilded mixed results. [12], [18] [19]. As a result, validity of the EICA hypothesis remains disputed. Considering these contradictory findings, Müller-Schärer et al. (2004) and Joshi and Vrieling (2005) proposed a more specific prediction by distinguishing specialist and generalist herbivores: introduced plants are expected to decrease the defense against specialist herbivores but increase the defense against generalist herbivores because they escaped from specialist herbivores but may still be attacked by generalist herbivores [20], [21]. Doorduin and Vrieling's (2011) metaanalysis supproted this prediction. Thus, further tests of the EICA hypothesis need to discriminate between the effects of specialist and generalist herbivores [22].

While the EICA hypothesis assumes the absence of herbivores in exotic ranges, this is not always the case. Native specialists may also invade exotic ranges where their host plants are already naturalized [4], [23], [24]. In addition, native specialists are often intentionally introduced to control or suppress invasive plants [25]. When a native specialist becomes present in the exotic range, an invasive plant population with reduced defense will suffer serious damage [26]. In this case, natural selection may favor genotypes with stronger defense against the specialist [27]. As a result, if the plant's defensive capacities could not be rapidly recuperated the population would be in danger of extinction. Despite the long history of biological invasion, we discovered only one study that has reported the evolutionary consequences of a plant's re-association with a coevolved herbivore [27], [28]. Using herbarium specimens spanning a 152 year period, Zangerl et al. (2005) found that the wild parsnips (*Pastinaca sativa* L.) had increased their toxic compounds after an accidental introduction of parsnip webworm (*Depressaria pastinacella* Duponchel)[28]. This provides a indication of invading plant species being able to restore defensive capabilities after the re-colonization of specialist herbivores. In this study, we use another approach to study ongoing recovery of defensive ability against a native specialist. We compared the geographical variations of defense level in interactions between the host plant *Ambrosia artemisiifolia* L (Asteraceae) and its specialist herbivore *Ophraella communa* LeSage (Coleoptera: Chrysomelidae).

Ragweed, *A. artemisiifolia*, is native to North America and was established in Japan more than 100 years ago [29]. By the 1950s, it was widely distributed throughout the Japanese islands [29]. In their native range, *A. artemisiifolia* populations are subject to attacks from a range of generalist and specialist herbivores [30]–[33]. However, naturalized populations of, *A. artemisiifolia* populations in Japan remained notably free from native enemies until the accidental introduction of a specialist herbivore, *O. communa*, in 1996 [34]. Since the introduction of *O. communa*, local *A. artemisiifolia* populations have been heavily damaged [35] and *O. communa* has rapidly expanded its distribution over the main Japanese islands of Honshu, Shikoku, and Kyushu [36]. However, *O. communa* has not yet colonized some of Japan's more remote islands. According to the EICA hypothesis, these remote *A. artemisiifolia* populations may lack defensive ability but achieve higher growth rate than the native plants, because specialist herbivores remain absent. In contrast, various *A. artemisiifolia* populations in mainland Japan have been subject to intensive herbivore attacks by introduced *O. communa* over periods from 11 to 13 years.

To study the evolutionary responses of *A. artemisiifolia* to the reassociation with its specialist herbivore, we conducted an experiment with *A. artemisiifolia* seeds and seedlings obtained from various populations, including native US regions as well as the main and more remote Japanese islands. We also conducted a bioassay experiment to clarify whether the growth of specialist herbivores diffesr on leaves of *A. artemisiifolia* obtained from native US regions, the main Japanese islands or more remote islands. From these experiments, we answer to the following questions. (1) Do *A. artemisiifolia* plants from enemy-free environments (i.e. the Japanese remote islands) show higher growth rate and lead to better growth performance of the specialist herbivore *O. communa* than the plants from native populations? (2) Do *A. artemisiifolia* populations after reassociation with *O. communa* (the Japanese main islands) lead to weaker growth performance of the specialist herbivore than the enemy-free populations?

## Results

### Plant growth

**Figure 1:**
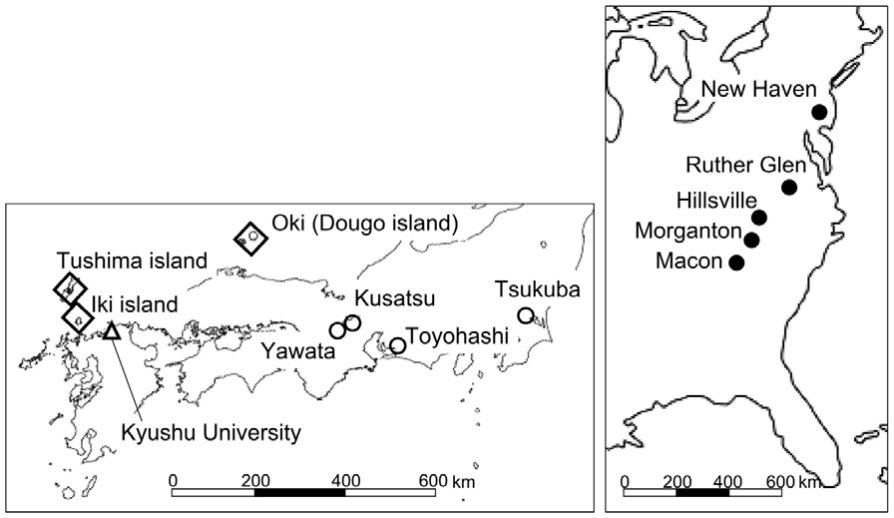
Plant height of *Ambrosia artemisiifolia* genotype from native (solid line) and Japanese remote islands (dashed line) at 40, 60, 70, 80 days after transplanting. Numbers of samples are 59 for Japanese remote islands and 99 for native population. Err bars are SE.

We compared *A. artemisiifolia* heights 40, 60, 70 and 80 days after transplantation (DAT). There were significant differences in *A. artemisiifolia* height between populations at a single site (F_7,148_ = 21.132, P<0.001). There was no significant difference in height 40 days after transplantation between introduced and native populations (χ^2^ = 0, p = 1). However, *A. artemisiifolia* from introduced populations grew higher than native populations at 60, 70, 80 DAT compared to height at 40 DAT (group×days interaction χ^2^ = 98.3, p<0.01, χ^2^ = 74.63, p<0.01, χ^2^ = 58.04, p<0.01 respectively, Fig. 1). During the period from 50 to 90 DAT, introduced populations exhibited a faster growth rate than native populations (Native: 0.86 cm· day^−1^, Introduced 1.37 cm·day^−1^).

### Bioassay

**Figure 2:**
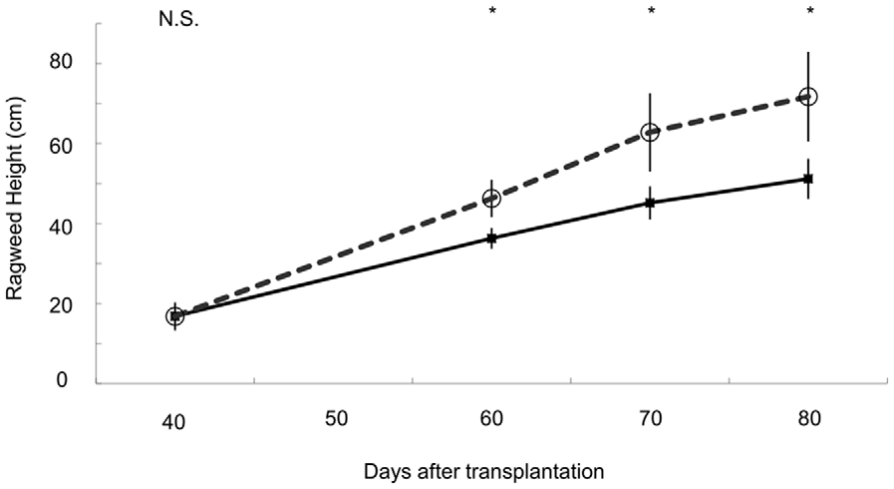
Specialist bioassay reared on *Ambrosia artemisiifolia* plants from Native, Japanese remote and Japanese main islands populations. Numbers under population name are sample size. Err bars are SE. (a) Larval survival of the *Ophraella communa* until pupation. (b) Mean days to pupation from hatching. (c) Dry weight of adult beetle soon after eclosion. In Figure 2a-c, three bars in the right side show the results pooled for the native area, the Japanese remote islands and the Japanese main islands. An asterisk indicates the statistically significant difference, P<0.01.

There were significant differences in days to pupation and *O. communa* dry weight between Tsukuba's 1998 population (see Materials and Methods) and Japanese remote islands populations (F_1,546_ = 39.405 P<0.001 for time to pupation, F_1,378_ = 7.8329 P = 0.005 for dry weight). Therefore, we separately analyzed *O. communa* raised on Tsukuba's 1998 and remote island populations in the analyses of the pupation time and the dry weight. *O. communa* reared on leaves coming from the Japanese remote islands populations showed significantly higher survival rates, shorter time to pupation and heavier adult dry weight than those fed on native host leaves (Table 1, Fig. 2). On the other hand, *O. communa* reared on plants belonging to the Japanese main islands populations showed significantly lower survival rate than remote island populations (Table 2, Fig. 2a). There was, however, no significant difference in the time to pupation and adult dry weight between remote and main islands populations (Table 1, Fig. 2b,c).

**Table 1:** Results of tests using GLMMs (Generalized Linear Mixed Models) for the survival rate of *Ophraella communa*, the time to pupation, and the dry weight fed on *Ambrosia artemisiifolia* plants originated from the populations of United States and Japanese remote islands.

		d.f	?2	P
Survival rate				
	Group	1	14.9	<0.001
Days to pupation				
	Group	1	13.6	<0.001
Dry weight				
	Group	1	10.79	<0.001

Chi-square values are obtained from likelihood ratio test.

**Table 2:** Results of tests using GLMMs (Genralized Linear Mixed Models) for the survival rate of *Ophraella communa*, the time to pupation, and the dry weight fed on *A. artemisiifolia* from the populations of remote and main islands in Japan.

		d.f	?^2^	P
Survival rate				
	Group	1	7.57	<0.01
Days to pupation				
	Group	1	1.8	0.18
Dry weight				
	Group	1	0.28	0.59

Chi-square values are obtained from likelihood ratio test.

## Discussion

Our results partially support the EICA hypothesis from both the host plant side and specialist herbivore side. Introduced *A. artemisiifolia* from Japanese remote islands exhibited a genetic predisposition for faster growth in height than native plants did. Likewise the specialist herbivore *O. communa* performed better on the Japanese *A. artemisiifolia* than on native plants. The difference in plant height between native and introduced populations was likely due to a higher allocation to growth at the expense of defense capacity. The temperature, which may influence the outcome of the common garden experiments [37], is unlikely to be the primary factor because mean temperatures in July in both the native and introduced sampling sites were similar and overlapping; 21∼24°C in the native and 23∼26°C in the introduced site (Weatherbase http://www.weatherbase.com/ and Japan Meteorological Agency http://www.jma.go.jp/jma/index.html). However, differences in other factors between sampling sites such as water availability and soil nutrients might have affected the outcome of the experiments. Hodgins and Rieseberg (2011) also reported that European populations of introduced *A. artemisiifolia* showed greater growth and reproduction compared to natives[38]. We found that the growth performance of *O. communa* was better when fed with the foliage from the introduced, enemy-free *A. artemisiifolia* (Japanese remote islands) in comparison to the foliage from the native populations. If *O. communa* was fed with the foliage of introduced *A. artemisiifolia* invaded by *O. communa* (Japanese main islands) the growth performance was worse than on the foliage from the enemy-free plants. These results suggest that the defense level of *A. artemisiifolia*, which is inferred to have decreased in enemy-free habitats, had been regained rapidly by populations after the reassociation with the native specialist *O. communa*. This recovery was likely to have occurred within 10–12 years since the introduction of *O. communa* in 1996, suggesting that plant defense capacity is evolutionarily very labile. Based on the EICA hypothesis, we consider that the higher defense level of the Japanese main islands populations is a direct result of herbivore attacks during the recent 10–12 years of renewed exposure. On the other hand, the differences between the remote and main islands populations could also be due to other differences attributable to the varying degree of isolation. First, populations in remote islands might maintain lower genetic variability than main islands populations due to bottleneck effects and/or restricted gene flow [39] and evolutionary recovery of defense against the renewed exposure to the specialist could be slower. Second, differences in native generalist herbivores between remote and main islands populations might influence the differences in defense level between them. The latter possibility is, however, less likely because very few native generalist herbivores have been observed in the introduced *A. artemisiifolia* populations [40].

These findings appear to be inconsistent with the results of an inter-continental reciprocal transplant experiment on the native and introduced *A. artemisiifolia* populations performed by Genton et al. (2005) [41]. This 2005 study compared the herbivore damage level in *A. artemisiifolia* individuals, comparing traits between native North American genotype and introduced French genotype in both native and introduced ranges. Although transplanted *A. artemisiifolia*, irrespective of the origin, suffered far less attacks by herbivores in France than in North America, they reported that the introduced *A. artemisiifolia* did not show any evidence of evolutionary loss of defense compared to the native *A. artemisiifolia*. They suggested that the toxins were retained in the French populations as a defense against generalist herbivores or as allelochemicals. However, it should be noted that the study by Genton *et al.* was mainly concerned with generalist herbivores and specialist herbivores were not in the focus of their field experiments. In contrast the EICA hypothesis focuses specifically on invading species that have been freed from the pressure of native specialist herbivores [12]. As there are fundamental differences between defense mechanisms against specialists and generalists [20], introduced plants may indeed fail to decrease their defense against the generalist herbivores in the introduced areas [20]–[22]. Therefore, it remains uncertain whether French and native *A. artemisiifolia* populations do indeed differ in their defense capabilities to specialist herbivores. In the present study, we found that plants originating from naturalized populations showed higher growth than plants from the native populations in a controlled environment. We also found that the growth performance of the specialist herbivore was lower when fed with the foliage from native plants in comparison to the foliage from invading plants. These results strongly suggest that the defense capacity of *A. artemisiifolia* has been influenced by the presence of specialist herbivores. However, it remains, uncertain whether the Japanese populations differ in their defense capacity against generalist herbivores compared to the native populations. The Japanese population might have reduced the defense capacity against generalist herbivore. Alternatively, they might maintain the defense capacity against generalist herbivore because only occasional damage by generalist herbivores are found in the introduced range of Japan [40]. Thus our results are not necessarily inconsistent with the finding of Genton et al. (2005), but rather suggest that further studies are required to distinguish effects of generalists and specialists on EICA more carefully.

In addition to the case of *A. artemisiifolia*, there are a number of studies that have tested the EICA hypothesis for various other plant species. Some studies have supported the hypothesis while others did not [42]. To explain such conflicting evidence, Dietz and Edwards (2006) proposed the importance of time since invasion [43]. Here they divided invasion periods between primary and secondary phases of invasion. They reasoned that the introduced species are initially subject to weaker competition, but in the secondary phase the competition becomes fierce once again, which is why the introduced species evolve higher competitive ability. The authors also argued, for species and populations they studied, that the data from the four species introduced into new areas 200–250 years ago revealed strong support for the EICA hypothesis, three of which had invaded closed, competitive native vegetation. On the other hand, the eight species showing no support to the EICA hypothesis had been introduced to the new areas 50–150 years ago and seven of them were noted to be restricted to disturbed habitats with more open vegetation. The results of this study conflicted somewhat with those of Dietz and Edwards (2006) because in this case *A. artemisiifolia* despite being introduced to Japan only around 100 years ago and becoming established in disturbed environments, still provided evidence in favor of EICA. Here we showed that the evolutionary response occurred even within a 13 year period, indicating that the evolution can act much faster than the time scale proposed by Dietz and Edwards (2006). As the speed of evolution depends on the generation time along with the interaction of natural selection and genetic variability, more careful comparisons of characteristics of host plants and herbivores in native and introduced ranges are required to generalize the conditions where EICA occurs as suggested by Atwood and Meyerson (2011)[44]. As for the strength of competition, it is difficult to test the idea that the introduced species are initially subject to weaker competition. However, we observed high seedling density of *A.artemisiifolia* in the field in spite that they grow in disturbed habitats, suggesting that the intraspecific competition rather than competition with the native vegetation predicted by Dietz and Edwards (2006) could be a major selective force for plant height.

Our study has implications for the biological control of invasive plants. It was suggested that if invasive plants decrease their defense against specialists in an introduced range, biocontrol agents could be more effective for use against an invasive genotype in comparison to the native genotype [20], [42]. *O. communa* reared on *A. artemisiifolia* from the Japanese populations taken in the areas where the specialist herbivores are absent achieved higher survival rate, shorter times to pupation and greater weight than the individuals reared on the native *A. artemisiifolia*. This appears to support such a possibility. However, our study also suggests that the *A. artemisiifolia* populations in the main islands have been evolving their defense against recently-introduced specialists. Although numerous studies have demonstrated that the evolutionary change can occur even within a few generations in natural populations [45], [46], little is known about the evolution of defense in target plants under the introduction of biocontrol agents [47], [48]. Our study, together with the findings of Zangerl and Berenbaum (2005), shows that the alien plants *Pastinaca sativa* can rapidly evolve defense against a specialist *Depressaria pastinacella* after the reassociation. This suggests that the effectiveness of a biocontrol agent may be weakened over time.

In conclusion, we found that the *A. artemisiifolia* naturalized in Japan had higher growth capacity than *A. artemisiifolia* from its native range. This was probably at the expense of defense capacity. We also found evidence that the naturalized *A. artemisiifolia* populations can quickly restore defense capacity after the renewed exposure to the native specialists. These results are consistent with the predictions of the EICA hypothesis. Such rapid evolution of defense capacity deserves more attention. As suggested by [49], the resurrection approach, using stocked ancestral seeds for experiments, is a promising way to understand contemporary evolutionary change.

## Materials and Methods

### Ethics statement

No specific permits were required for the described field study. The insect and plant species collected are not endangered or protected.

### Study species

Common ragweed, *A. artemisiifolia*, native to North America, is a vigorous invasive weed and naturalized to many places over the world [50]. It is an annual which germinates in spring and produces seeds in late summer. In both native and naturalized ranges, *A. artemisiifolia* often grows in habitats that have been disturbed by human activity such as the urban areas and roadsides in the rural areas [30]. *Ambrosia artemisiifolia* is particularly notable for its link to human health as its wind-dispersed pollens can cause many allergic responses[51].

*Ophraella communa* (Chrysomelidae) was also native to North America and accidentally introduced to Chiba prefecture, Japan in 1996 [34]. After the initial introduction, *O. communa* rapidly expanded its range and became established in most of the Japanese islands [36]. Overwintering adults deposit a cluster eggs on the host plant in spring. At 25°C, three weeks are required from hatching to adulthood. Three or four generations annually occur before diapause in late autumn. In its native range, *O. communa* uses several species of *Ambrosia* and *Iva* as host plants [31]. In Japan, *O. communa* mainly forage upon *A. artemisiifolia* in its early growing season but also has used giant ragweed *Ambrosia trifida* (also native to North America) in cases where *A. artemisiifolia* plants had become defoliated completely by *O. communa* [35]. *O. communa* is foliivorous but often feed on the reproductive parts of plant. *Ophraella communa* has also been considered as a potential biocontrol agent to control *A. artemisiifolia* in Australia and Eastern Europe [52], [53].

### Plant growth

**Figure 3:**
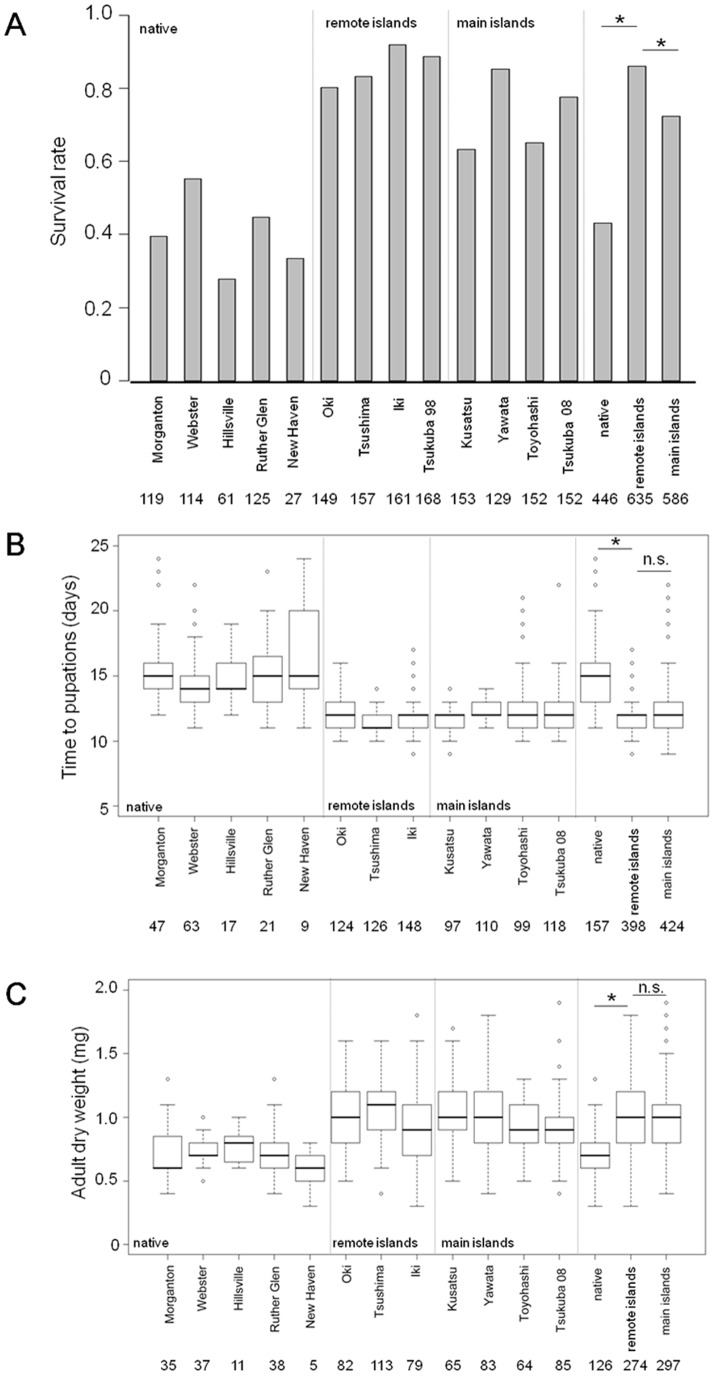
Locations of seed and seedling source populations of *Ambrosia artemisiifolia* in the United States (shaded circle), and Japan (un-shaded diamond for remote islands populations, un-shaded circle for mainland populations). The experimental site is represented as an un-shaded triangle.

In 2008, we collected seeds of *A. artemisiifolia* from five populations in the United States (Webster, Morganton, Hillsville, Ruther Glen, New Haven) and from three populations located in the Japanese remote islands (Tsushima, Iki, Oki). We also collected seeds in Honshu, Japan's largest island, in 2008 (Tsukuba) and 2009 (Kusatsu, Yawata, Toyohashi). The Honshu populations had experienced *O. communa* attacks for at least the past 11years. The Tsukuba population had experienced the same for 13 years [36]. At each site, seeds were collected from about ten individuals (Fig. 3). The collected seeds were stored at 4°C until the experiment. In addition, we obtained seeds that were collected in Tsukuba in 1998, shortly after the introduction of *O. communa*. In both cases, seeds were harvested in bulk, and we could not examine possible effects of maternal plants. In 2009, we sowed 200 seeds from each population in the germination beds filled with commercially available garden soil (Sun&Hope Co., Japan) that is a mixture of sand, pumice, volcanic ash, humus and fertilizer and free from other seeds. At about 10 days after germination we randomly selected 20 plants from each population to be individually transplanted to pots filled with 3 liters of garden soil. This was because days to germination differed among the population. In addition to seedlings grown from seeds, 20 seedlings from each of the field populations of Kusatsu, Yawata, and Toyohashi in Honshu were directly transplanted to pots and placed in the meshed house at Kyushu University Fukuoka prefecture, Japan soon after field sampling. We grew all plants in a mesh house to prevent insect damage. Plants were watered every day. Plant height was measured once a week from June to August, until plants were harvested for the bioassay experiment. Plants grown from seedlings were not used for height growth analysis due to the initial size effect and were therefore only used for the bioassay experiment, although these plants grew in the same way as plants grown from seeds.

### Bioassay

To evaluate the differences in defense levels of each *A. artemisiifolia* population against the specialist herbivore, we measured the fitness components of *O. communa* fed on each A. artemisiifolia population. About 3000 *O. communa* adults were collected not from *A. artemisiifolia* but from *A. trifida* populations near Kyushu University and reared in the laboratory because *O. communa* defoliated many *A.artemisiifolia* individuals and began to use A. trifida populations when we started the experiment. After hatching, we divided first-instar larvae into a few groups and reared them on leaves of *A. artemisiifolia* taken from different populations that were grown in the mesh house of the Kyushu University. Every two or three days, we replaced the leaves and counted the number of surviving larvae. To record the days to pupation, we checked larvae everyday from 10 days post-hatching. Emerged adults were collected after eclosion and their weight was measured after drying in an oven for more than 6 hours at 60°C, (sufficient to completely dry adults of *O. communa.*).

### Statistical analyses

To compare the height of *A. artemisiifolia* derived from the native and introduced populations at 40 days after transplantation, we employed Generalized Linear Mixed Models (GLMMs) with groups (native and introduced populations), and populations (nested in groups) as random effects with the Gaussian distribution and identity link. To compare the growth rate between native and introduced populations, we also employed GLMMs with groups (native and introduced populations), days after transplantation, their interactions as explanatory variables and ID and populations (nested in groups) as random effects with the Gaussian distribution and identity link. ID was included as a random effect because we measured height of the same individuals at different time points. We used the Bonferroni procedure to correct for multiple comparisons.

To examine the EICA hypothesis and the possibility of rapid recovery of defense, bioassay experiments were analyzed in two categories (native vs. Japanese remote islands and Japanese remote islands vs. main islands). We also employed GLMMs to analyze the data from bioassay experiments and considered groups as explanatory variable, populations (nested within groups) and *O. communa* lineage (same egg cluster) as random effects. Survival rates were analyzed with a binominal distribution and a logit link function since the response variable was binary. Days to pupation and adult dry weights were analyzed with a Gaussian distribution and identity link functions. We used the likelihood ratio test for overall significance of the explanatory variables. The interactions were excluded from the analyses of the bioassay data. In all analyses, we examined the difference between remote island populations and Tsukuba's 1998 population that experienced herbivory of *O. communa* for only 1 or 2 years. If the difference was insignificant, we included the 1998 population with the remote island populations and reanalyzed data between sites. For all statistical analyses, we used the software R [54] with the package lme4 and the function lmer.
