# Correction: Changes in Defense of an Alien Plant *Ambrosia artemisiifolia* before and after the Invasion of a Native Specialist Enemy *Ophraella communa*

**DOI:** 10.1371/annotation/868d00f2-375e-421f-8435-0e628c0567bd

**Published:** 2012-11-12

**Authors:** Yuya Fukano, Tetsukazu Yahara

The images for Figures 2 and 3 were incorrectly switched. The image that appears as Figure 2 should be Figure 3, and the image that appears as Figure 3 should be Figure 2. The figure legends appear in the correct order. 

